# Ontogenetic changes in the body structure of the Arctic fish *Leptoclinus maculatus*

**DOI:** 10.1038/s41598-023-30251-5

**Published:** 2023-03-06

**Authors:** S. N. Pekkoeva, E. A. Kondakova, S. Falk-Petersen, J. Berge, S. A. Murzina

**Affiliations:** 1 Institute of Biology, Karelian Research Centre of the Russian Academy of Sciences (IB KarRC RAS), 185910 Pushkinskaya, 11, Petrozavodsk, Republic of Karelia Russia; 2 Biological Faculty, St. Petersburg State University, University Embankment 7/9, St. Petersburg, 199034 Russia; 3 Federal State Scientific Establishment “Berg State Research Institute on Lake and River Fisheries” (GosNIORH), St. Petersburg Branch of VNIRO, Russian Federal Research Institute of Fisheries and Oceanography, Makarova Embankment 26, St. Petersburg, 199053 Russia; 4 Akvaplan-niva AS, Fram Centre, 9296 Tromsø, Norway; 5 Department of Arctic and Marine Biology, UiT The Arctic University of Norway, 9037 Tromsø, Norway; 6 The University Centre in Svalbard, 9171 Longyearbyen, Norway; 7 Centre for Autonomous Marine Operations and Systems, Norwegian University of Science and Technology, Trondheim, Norway

**Keywords:** Developmental biology, Ecology, Structural biology

## Abstract

Histological studies of the ontogenetic changes in Arctic marine fishes are often fragmented and incomplete. Here we present a comprehensive histological ontogenetic analysis of the daubed shanny (*Leptoclinus maculatus*) from the Arctic, characterizing its development as it undergoes a series of changes in the organ and tissue organization, especially during the postlarvae transition from the pelagic to benthic lifestyle. The thyroid, heart, digestive tract, liver, gonads, blood, and the lipid sac of the postlarvae at different developmental stages (L1–L5) were studied for the first time. We found that *L. maculatus* has structural characteristics of marine fish developing in cold, high-oxygen polar waters. We conclude that the presence of the lipid sac and the absence of distinguishable red blood cells in pelagic postlarvae are unique features of the daubed shanny most likely linked to its successful growth and development in the Arctic environment.

## Introduction

The arcto-boreal fish *Leptoclinus maculatus* (Fries, 1838), daubed shanny, belonging to the family Stichaeidae is a common species of polar ecosystems of high ecological importance^1–3^. It spawns during winter and lives as a pelagic post-larva up to 5 years before it turns to a benthic lifestyle as a juvenile^1^. The daubed shanny has a small body size (adults are up to 20 cm in length), wherein the lipids account for 40% of its dry mass and are stored in lipid droplets between muscle fibers in young fish^4,5^. The postlarvae have a unique adaptive provisory organ, the lipid sac, made up of closely packed lipid-filled compartments^4,5^. It is only presented in pelagic postlarvae stages with lipid content comprising up to 92% of its dry weight^6^. The lipid sac functions as an energy storage organ, possibly also providing for the buoyancy control and / or serving as a disruptive camouflage strategy. Hence, its capability to store large amounts of energy-rich lipids makes *L. maculatus* a potentially important prey species for many predators in the Arctic marine food web (fishes, birds, and mammals)^1,3,6–8^.

Morphological ontogenetic features of marine bony fish species such as the trailing gut in mesopelagic myctophids, melanostomids and other, external intestinal loops in bythitid, elongated fin rays in carapid fishes are known as transient adaptative structures in larvae^9–12^. The lipid sac of the daubed shanny postlarvae is considered to be a unique morphological trait among the Arctic marine fishes^4^. When the fish mature and settle in muddy caves at the sea floor, the lipid sac is reduced and eventually gets lost^1,6,13^. Biochemical analysis showed changes in the lipid content of the muscles and the lipid sac during the daubed shanny postlarval development^6,13^, whereas the detailed information about its anatomy and histology may significantly advance our understanding of structure–function features during the postlarvae ontogenetic stages.

The aim of the present research was to map and characterize the ontogenetic changes of *L. maculatus* during postlarval development in relation to its life history. To the best of our knowledge, this is the first study to provide a histological analysis of organs and tissues (based on the serial sagittal sections of the whole body) of postlarvae at different developmental stages. The histological description of ontogeny in high- latitude polar marine fish species are generally missing or largely fragmented, although they may shed light on fish life cycle and behavior in the Arctic environment.

## Materials and methods

There are several stages of the daubed shanny postlarvae development—pelagic L1, L2, L3, transitional—L4/L4* and demersal L5—early juvenile, which differ in size, weight, color and body pigmentation, condition of lipid sac according to the classification by C. Mayer Ottesen et al.^1^ and the data from our research^6,14^. The structure of organs and tissues (thyroid, heart, liver, digestive tract, gonads, blood, the lipid sac) of *L. maculatus* postlarvae at the stages L1, L2, L3, L4* and L5, were studied. The postlarvae were collected from the research vessel «*Helmer Hanssen*» (UiT The Arctic University of Norway) in fjords of the Svalbard archipelago (Kongsfjord and Rjipfjord). The fishes were fixed in 4% formaldehyde solution (buffered). The material was washed from the fixative in phosphate-buffered saline (PBS), dehydrated in alcohols of ascending concentration, and embedded in Paraplast (Leica, Germany) according to the standard procedure. Prior to embedding the tails and pectoral fins at L1–L3, as well as tails, heads, pectoral fins, and dorsal part of the fish body at the stages L4* and L5 were removed. Serial sagittal sections 6–7 µm thick were obtained using a Leica SM 2010R sledge microtome (Leica Microsystems, Germany). The sections were stained with hematoxylin Carazzi's and eosin (Biovitrum, Russia). The preparations were viewed and photographed using a Leica DMI6000 microscope (Leica, Germany). The measurements were made using a Fiji software^15^. The length of the lipid syncytial nuclei (LSN) of the formed lipid sac, the long and short diameters of the thyroid follicles and the height of the thyrocytes in the thyroid gland were measured in postlarvae sectioned in sagittal plane at different stages of the postlarvae development. The number of studied lipid sacs and the thyroid glands and the measurements of their inner structures at different developmental stages of postlarvae were provided in the Tables 1 and 2. In order to exclude a risk of repeated measurement of the same nucleus in the syncytium of the lipid sac, at least four sections were left between the measured ones. Thyroid follicles were measured if the area of their maximum long and short diameters could be determined on serial sections. The height of thyrocytes was measured once for each follicle. The results were statistically processed using the R-programming language with basic packages^16,17^. Method of mixed linear models was used to confirm the assumption of overlapping ranges of the standard deviation (sd) of the variance for each fish within the developmental stage. The analysis was carried out using the "lme4" package in the R programming language. The confirmation of this hypothesis allowed to combine the measurements of inner structures of the studied fishes in one sample (averaging the values is one of the ways to get rid of samples from dependence) for each stage of development and perform a comparison of the studied parameters between different developmental stages. After checked for normal distribution Wilcoxon-Mann–Whitney test was used for this comparison. The study was based in the Resource Centre for Molecular and Cell Technologies (RC MCT) of the St. Petersburg State University and in the Scientific Center collective usage platform of the Karelian Research Centre of the Russian Academy of Sciences (KarRC RAS).

**Table 1:** Lengths of nuclei in lipid syncytial layer of the lipid sac of *L. maculatus* postlarvae at different developmental stages.

Stage of postlarval development	L1	L2	L3	L4*	L5
Number of the studied lipid sacs	4	2	2	2	2
Total number of measured nuclei	260	185	161	78	250
Length of nuclei, µm	6.29 ± 0.11	8.12 ± 0.17^a^	9.47 ± 0.21^ab^	16.58 ± 0.56^abc^	15.94 ± 0.3^abc^
Min–max length of nuclei, µm	3.33–15.71	4.14–15.72	3.08–17.33	7.64–32.72	5.68–39.88

Data are presented in form M ± SE.

^a^Differences in nuclei length were significant in comparison with the stage of development L1.

^b^Differences in nuclei length were significant in comparison with the stage of development L2.

^c^Differences in nuclei length were significant in comparison with the stage of development L3.

**Table 2:** The diameters of follicles and heights of thyrocytes in thyroid gland of *L. maculatus* postlarvae at different developmental stages.

Stage of postlarval development	L1	L2	L3
Number of the studied fish organs	3	2	2
Total number of measured thyrocytes	117	32	32
Height of thyrocytes, µm	5.62 ± 0.11	4.38 ± 0.16^a^	4.93 ± 0.16^ab^
Total number of measured follicles	186	63	60
Long diameter of follicles, µm	40.77 ± 1.31	63.13 ± 2.8^a^	53.71 ± 2.72^ab^
Short diameter of follicles, µm	28.82 ± 0.96	46.50 ± 1.99^a^	35.63 ± 1.97^ab^

Data are presented in form M ± SE.

^a^Differences in short or long diameters of follicles or height of thyrocytes were significant in comparison with the stage of development L1.

^b^Differences in short or long diameters of follicles or height of thyrocytes was significant in comparison with the stage of development L2.

The study was performed according to and within the regulations enforced by the Norwegian Animal welfare authorities and no specific permissions were required. All the experiments described in the present study and involving handling of animals were performed in full compliance with relevant international guidelines, including the ARRIVE guidelines, and regulations for the care and use of animals. Young fish were euthanized by rapid cooling followed by exposure in ice-cooled water and after that fixed in formaldehyde solution. The animal study protocol was approved by the Institutional Review Board (or Ethics Committee) of the Institute of Biology KarRC RAS (protocol code 168, Sept 28, 2021).

### Ethical standards

The authors declare to have no conflict of interest and that all applicable institutional, national, or international guidelines for the use and care of animals were strictly followed in the present study.

## Results

It was found that the dispersion of measurements (for the parameters as the length of LSN in the lipid sac, the height of the thyrocytes, the long and short diameters of the follicles in the thyroid gland) between individual fish within developmental stages are overlapped (Supplementary Materials, Fig. S1–S4 online). The hypothesis was confirmed using the method of mixed linear models. During the statistical analysis, a mixed model was selected with a fixed factor "Stage" and a random factor "Fish", which describes interindividual variability. To test hypotheses in the mixed models, likelihood-ratio tests (LRT) were used, since the LRT distribution is approximated by the χ^2^ distribution with df = dfM2 − dfM1. The detailed results were presented in Supplementary Materials, Results Section. Based on the statistical results of the mixed linear models and on the comparative range of the standard deviation (sd) of individual fish within the grouping variables (developmental stages), the observations were combined in one sample (Table 1—line “Total number of measured nuclei”; Table 2—line “Total number of measured thyrocytes” and “Total number of measured follicles”) for subsequent pairwise comparison between developmental stages.

## Lipid sac

### L1 stage, pelagic

**Figure 1:**
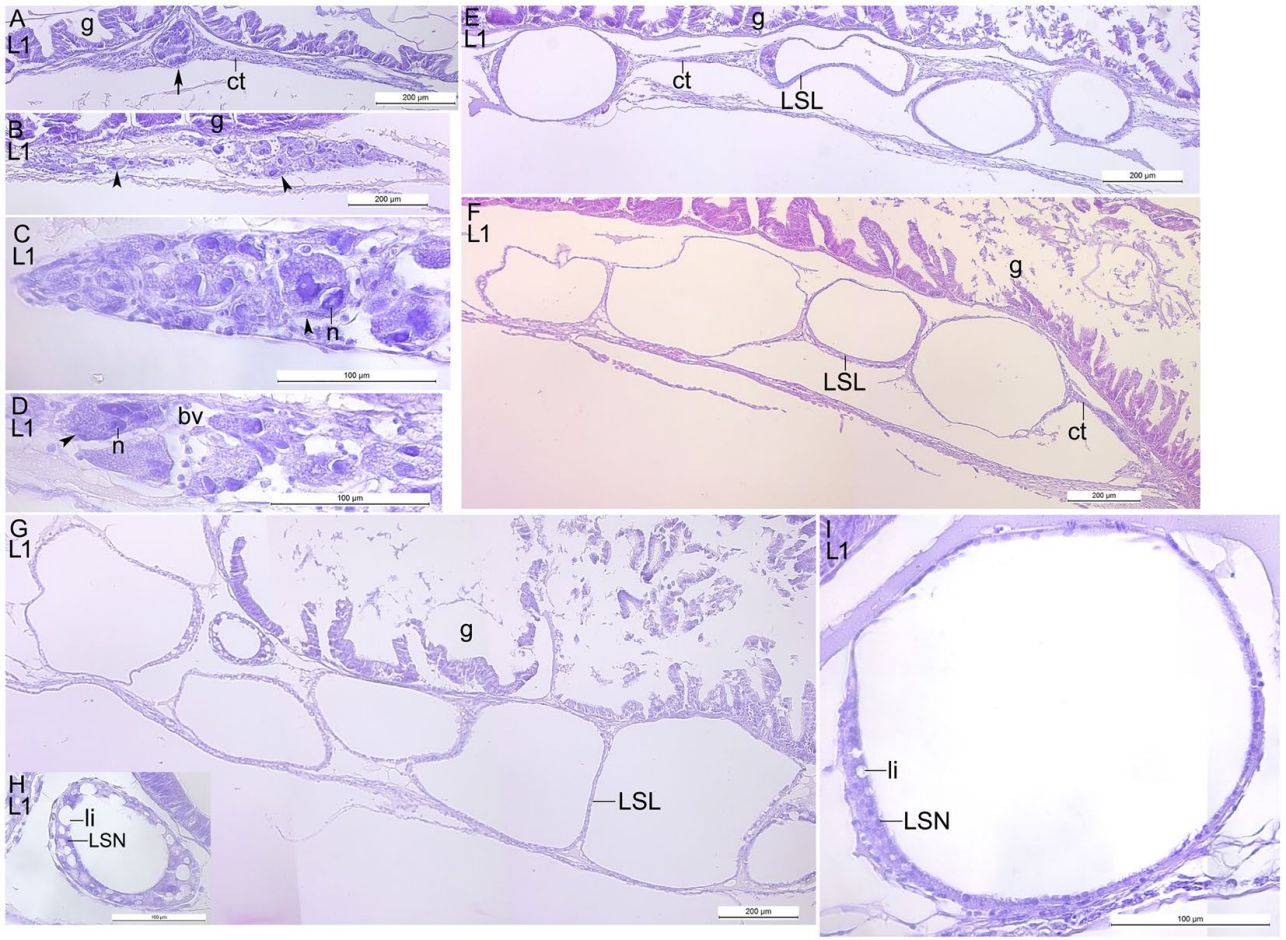
The structure of the lipid sac of *L. maculatus* at the L1 stage of development. Sagittal section. (**A**) The syncytial unit within the connective tissue. (**B–D**) The large multinucleate cells within connective tissue under the gut. (**E**) The lipid-filled units of immature LS. (**F**) The LS at the more advanced stage compared to “(**E**)”. (**G**) The more mature LS compared to “(**F**)”. (**H**) The region of “(**G**)” at the higher magnification. (**I**) The lipid-filled unit of the same specimen as in “(**E**)”. *g *gut, *ct *connective tissue, *n *nucleus, *bv *blood vessel, *LSL *lipid syncytial layer, *li *lipid inclusion, *LSN *lipid syncytial nucleus. An arrow indicates the syncytial unit, the arrowheads point to the large cells.

The early stage of the lipid sac development was studied. The multinucleate structures or large cells with more than one nucleus were located within the connective tissue under the gut (Fig. 1A–D). In the four studied postlarvae the lipid sac was immature (Fig. 1E–I).

### L2, L3 stage, pelagic

**Figure 2:**
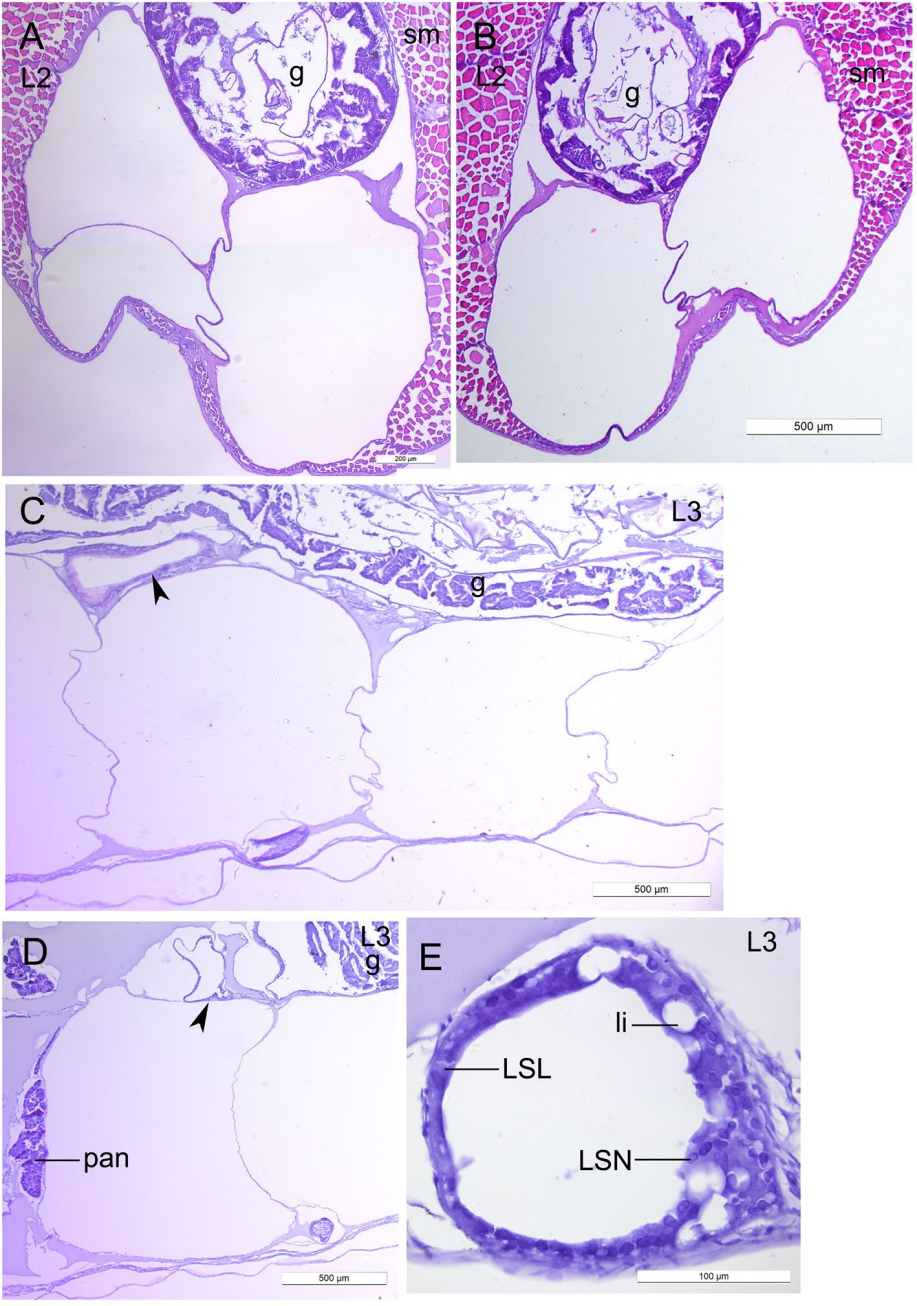
The structure of the lipid sac of *L. maculatus* postlarvae at the L2 and L3 stages of development. (**A,B**) Transverse sections of the lipid sac at the L2 stage, the two levels along AP axis. (**C,D**) Sagittal sections of the lipid sac at L3, showing lipid-filled units in proximity the gut and pancreas. Arrowheads point to the relatively small units. (**E**) Sagittal section of the thick-walled small unit with lipid inclusions in the wall and numerous lipid syncytial nucleus. *g *gut, *sm *skeletal musculature, *pan *pancreas, *li *lipid inclusion, *LSL *lipid syncytial layer, *LSN *lipid syncytial nucleus.

**Figure 3:**
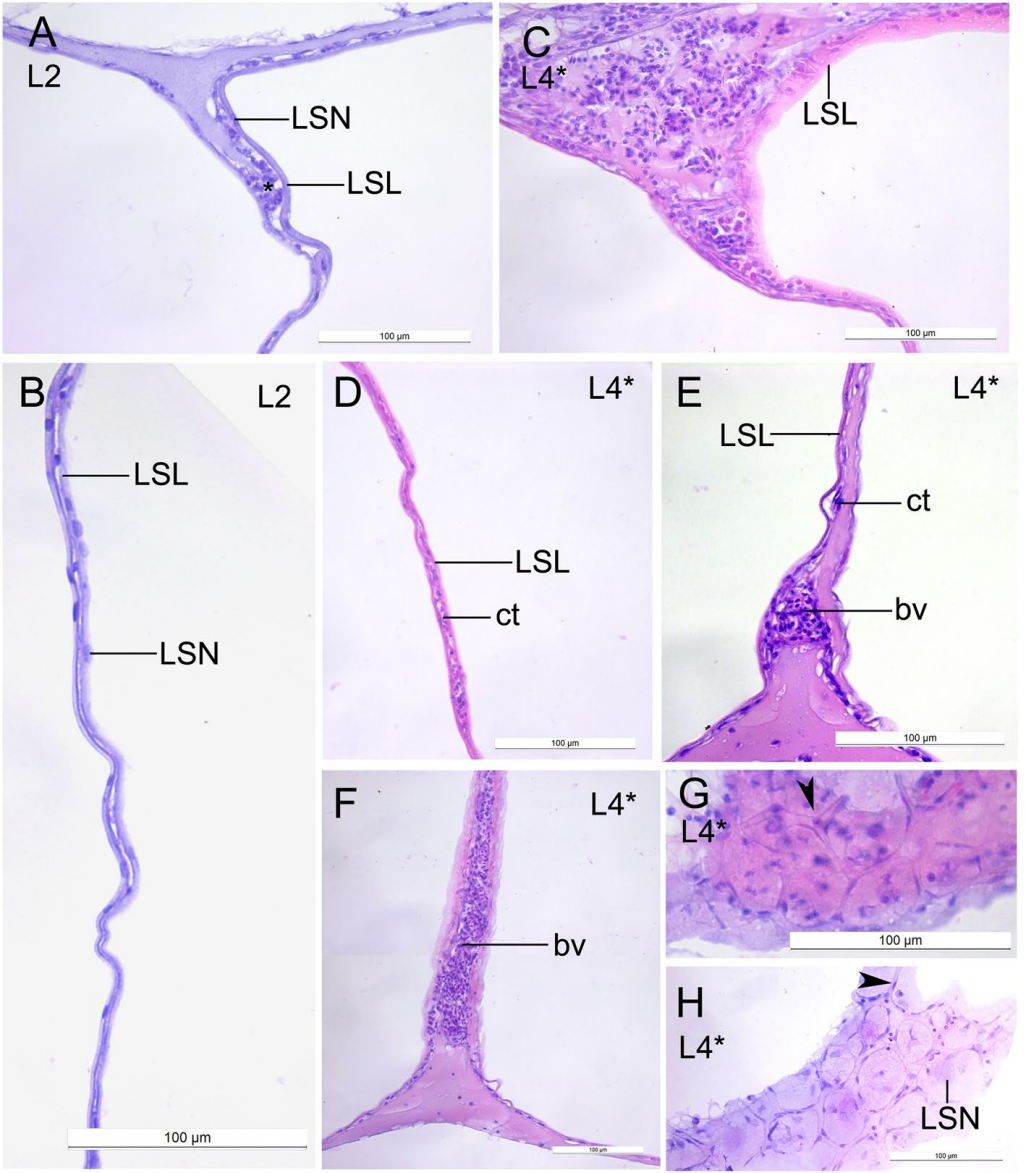
Structure of the lipid sac walls in *L. maculatus* at the most contracting the L2 and the L4* stages. Sagittal sections. (**A,B**) Lipid sac walls at the L2 stage. Connective tissue is indicated with asterisk. (**C**) The space between the units and its wall, blood vessels with red blood cells and connective tissue. (**D**) The thin lipid sac wall. (**E,F**) The walls with large blood vessels. (**G,H**) Sections through the walls of units. Arrowheads point to the blood vessels, forming network of polygons. *LSL *lipid syncytial layer, *LSN *lipid syncytial nucleus, *ct *connective tissue, *bv *blood vessel.

**Figure 4:**
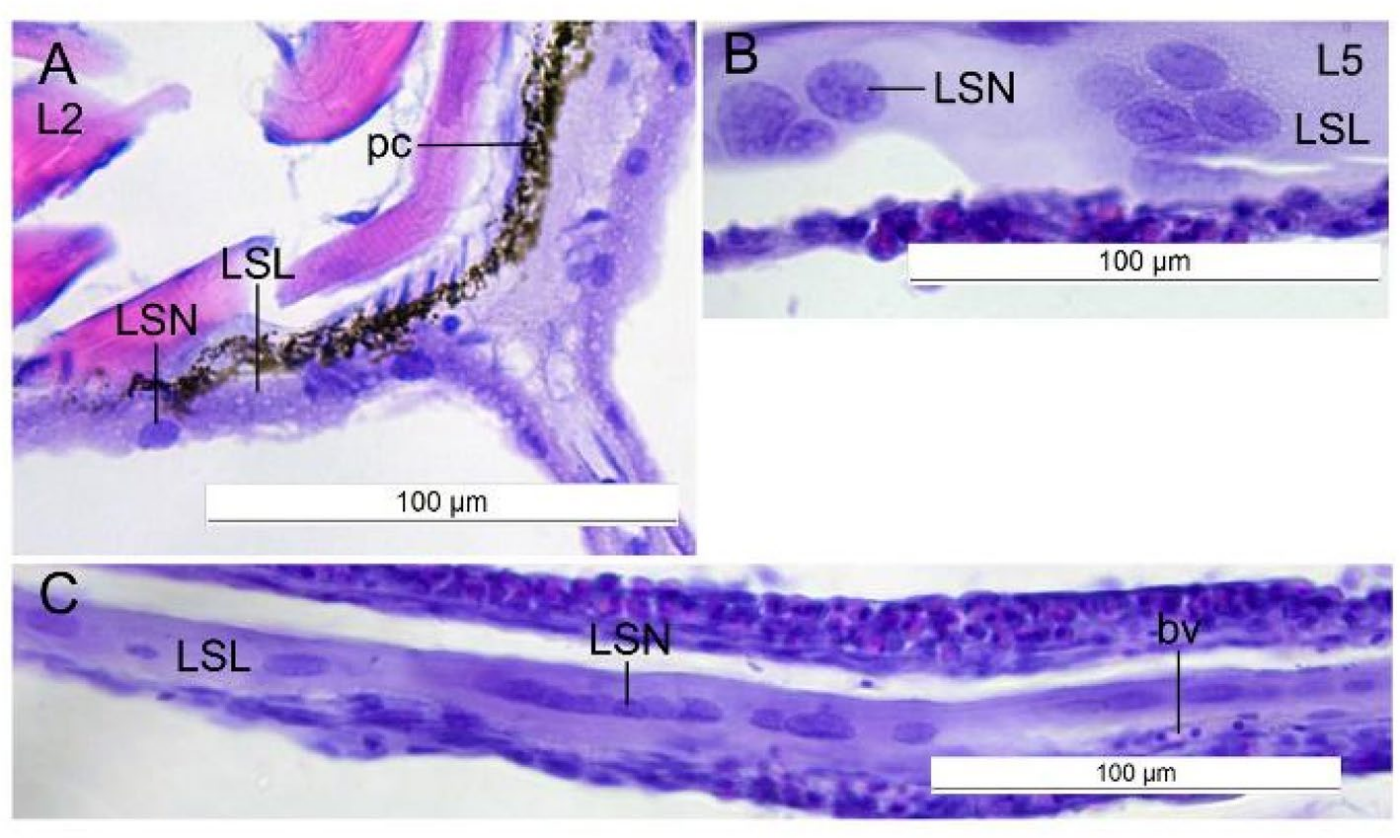
The lipid syncytial nucleus of *L. maculatus* postlarvae at the L2 and the L5 stages of development. Sagittal sections. (**A**) Relatively small elliptical lipid syncytial nucleus at the L2 stage. (**B,C**) Groups and rows of the enlarged lipid syncytial nucleus at the L5 stage. *LSL *lipid syncytial layer, *LSN *lipid syncytial nucleus, *pc *pigment cell, *bv *blood vessel.

The lipid sac was fully formed and the lipid sac units were gradually filled with lipids. The lipid sac compartments were situated under the gut as nearly bilateral rows, sometimes located one above the other (Fig. 2A,B). The lipid-filled units were in the proximity of the gut and pancreas (Fig. 2C,D). The spaces between the compartments and between them and the neighboring structures were filled with uniformly stained matrix. The wall of each lipid sac unit consisted of a connective tissue (up to several rows), blood vessels, and the multinucleate lipid syncytial layer** (**LSL), which interacts with lipids directly (Figs. 2E, 3A–F). The LSL of the large lipid compartments was generally thin, however, there were occasional local thickenings with unstained lipid inclusions in the cytoplasm. Sections through the walls showed that the blood vessels form the network of polygons (Fig. 3G,H). The LSN were numerous and mainly rounded or elliptical (Fig. 4A). The average lengths of nuclei of the lipid sac were presented in the Table 1. The measured LSN lengths at different stages were supplemented to the study^3^.

### L4* stage, transitional

The length of LSN significantly increased during the fish growth and especially during the transition period (from 9.47 at the L3 stage until 16.58 at the L4* stage) (Table 1) (L1 n = 260 vs L2 n = 185, W = 12,483, p-value < 2.2e−16; L2 n = 185 vs L3 n = 161, W = 10,239, p-value = 5.338e−07; L3 n = 161 vs L4* n = 78, W = 1093.5, p-value < 2.2e−16; L4* n = 78 vs L5 n = 250, W = 10,558, p-value = 0.2697). The mitotic figures, nuclear fragmentation, the pycnotic LSN or nuclei with any signs of destruction were not found.

### L5, demersal

The volume of lipid compartments decreased, and, on the contrary, the LSL thickness increased. Groups and rows of the enlarged LSN were found (Fig. 4B,C).

### Thyroid

The follicles of the thyroid gland (see Supplementary Fig. S5 online) of pelagic postlarvae were located diffusely in the branchial region near the ventral aorta. The colloid without reabsorption vesicles at the periphery was stained weakly. The follicles were mainly elliptical, but several triangular and irregular-shaped ones were also found (Fig. S5C,F,G). The chains of two, three and more small follicles and the pairs of conjoined follicles were observed (see Supplementary Fig. S5D–F). The height of thyrocytes decreased from the L1 to the L2 postlarval stage (L1 n = 117 vs L2 n = 32, W = 2951, p-value = 6.179e−07). The size of follicles increased from the L1 to the L2. The differences between the L1 and L2 stages were shown for long follicle diameters (L1 n = 186 vs L2 n = 63, W = 2524; p-value = 1.491e–11) and short follicle diameters (L1 n = 186 vs L2 n = 63, W = 2247; p-value = 2.683e−13) (Table 2).

### Heart

The heart of postlarvae was described from anterior to posterior (see Supplementary Fig. S6 online). The bulbus arteriosus (BA) was pear-shaped at all the studied stages. Its wall was subdivided into four layers: endocardial (endothelial), subendocardial, middle, and external (terms of Icardo et al., 1999). The inner surface of BA at the L1–L3 had compact ridges of relatively simple shape. The endocardial cells were generally convex (Fig. S6A). The daubed shanny had a single row of the conus valves of two pocket-like leaflets and the supporting sinus (Fig. S6B). The leaflets had an enlarged proximal body and a flap-like distal region (Fig. S6B). The ventricular myocardium was entirely trabeculated and lacked compacta (Fig. S6B–E). The atrioventricular ring myocardium was relatively thin, compacted and was continuous with the muscles of atrium and ventricle (Fig. S6D). The leaflets of atrioventricular (AV) valves were delicate (Fig. S6D). The thin-walled atrium with a network of trabeculae was located dorsally to the ventricle and partly encircled it. The sinus venosus was thin walled (Fig. S6E).

### Blood

Erythrocytes were absent from the blood vessels in the studied pelagic stages of postlarvae. They became prominent only by the L4* stage (see Supplementary Fig. S7 online).

### Digestive system

**Figure 5:**
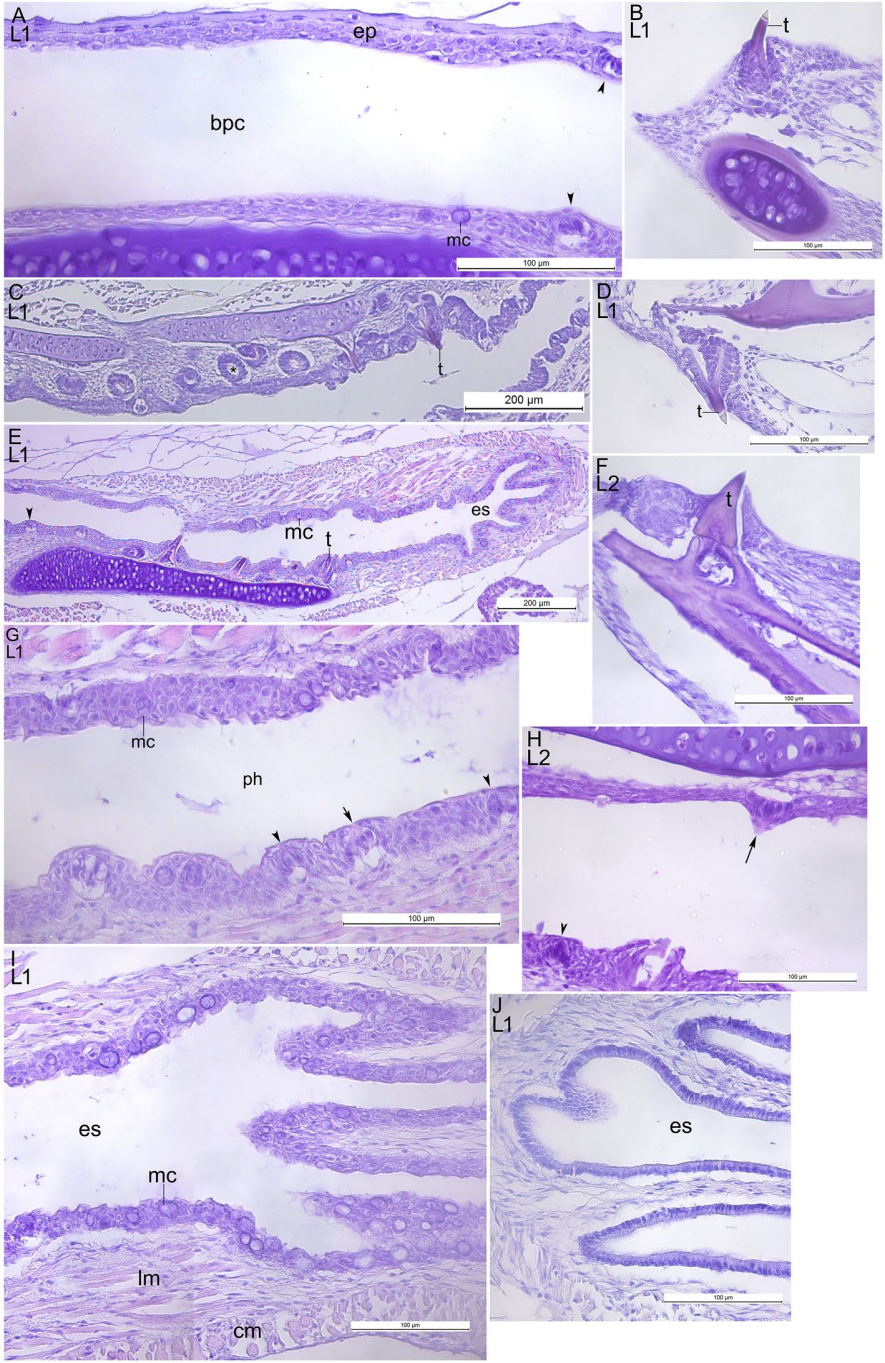
The digestive system of *Leptoclinus maculatus* postlarvae at the L1 and the L2 stages of development. (**A**) Buccopharyngeal cavity at the L1 stage. (**B**) The erupted lower jaw tooth at the L1 stage. (**C**) The upper pharyngeal teeth at the L1. (**D**) The erupted upper jaw tooth at the L1. (**E**) The lower pharyngeal teeth at the L1. (**F**) The lower jaw tooth at the L2 stage. (**G**) The taste buds in the pharynx at the L1 stage. (**H**) The taste buds at the L2 stage. Note the prominent taste buds with open receptor area. (**I,J**) The esophagus with longitudinal folds. The stratified epithelium with numerous mucus cells changes to the simple columnar epithelium posteriorly. *bpc* buccopharyngeal cavity, *ph* pharynx, *ep *epithelium, *mc *mucus cell, *t* tooth, *es *esophagus, *lm *longitudinal muscles, *cm *circular muscles. The immature and mature taste buds are indicated with arrowheads and arrows respectively. The tooth primordium is indicated with asterisk.

**Figure 6:**
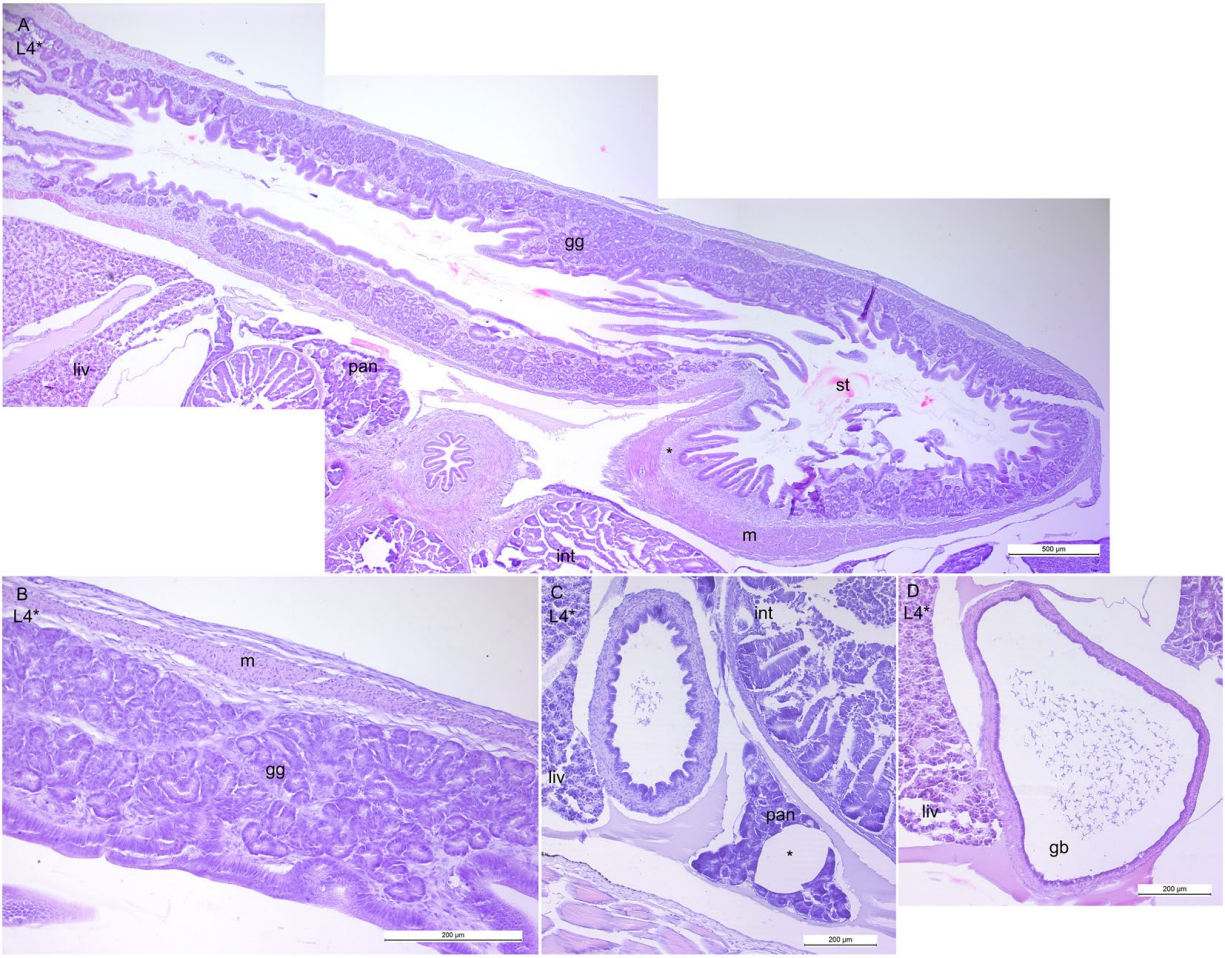
The digestive system of *Leptoclinus maculatus* postlarvae at the L4* stage of development (**A**) The stomach with glandular and aglandular (asterisk) regions. (**B**) The region of “(**A**)” at higher magnification showing gastric glands. (**C**) Duct of the pancreaticobiliary system and pancreas with adipose tissue (asterisk). (**D**) The gall bladder. *gg *gastric glands, *m *muscles, *pan *pancreas, *liv *liver, *int *intestine, *st *stomach, *gb *gall bladder.

The epithelium of the buccopharyngeal cavity in the postlarvae at the L1-L3 stages was stratified, of unequal thickness with single mucus cells (Fig. 5A). The jaw and pharyngeal teeth were at the different stages of development (Fig. 5B–F). Both upper and lower pharyngeal as well as lower jaw teeth erupted at the L1 stage. The taste buds were rare. The pear or onion-shaped taste buds with open receptor areas were observed at the L1 stage (Fig. 5G). They became more prominent at the L2 (Fig. 5H). The esophagus was lined with stratified epithelium with numerous mucus cells (Fig. 5I). In the distal region of the esophagus, the mucosa consisted of simple columnar epithelium without mucus cells (Fig. 5J). No gastric glands were seen during the L1–L3 stages. The intestinal mucosa was folded. Figure 6C,D show the hepatobiliary duct and the gall bladder at the L4* stage. The stomach with prominent glandular and aglandular regions was formed by the L4* stage (Fig. 6A,B). At this stage the pancreas contained adipose tissue (Fig. 6D).

### Liver

Small lipid inclusions were observed in the hepatocytes at the L1 stage; they were more prominent at the L2 and the L3 stages and decreased by the L4* stage (see Supplementary Fig. S8 online).

### Gonads

**Figure 7:**
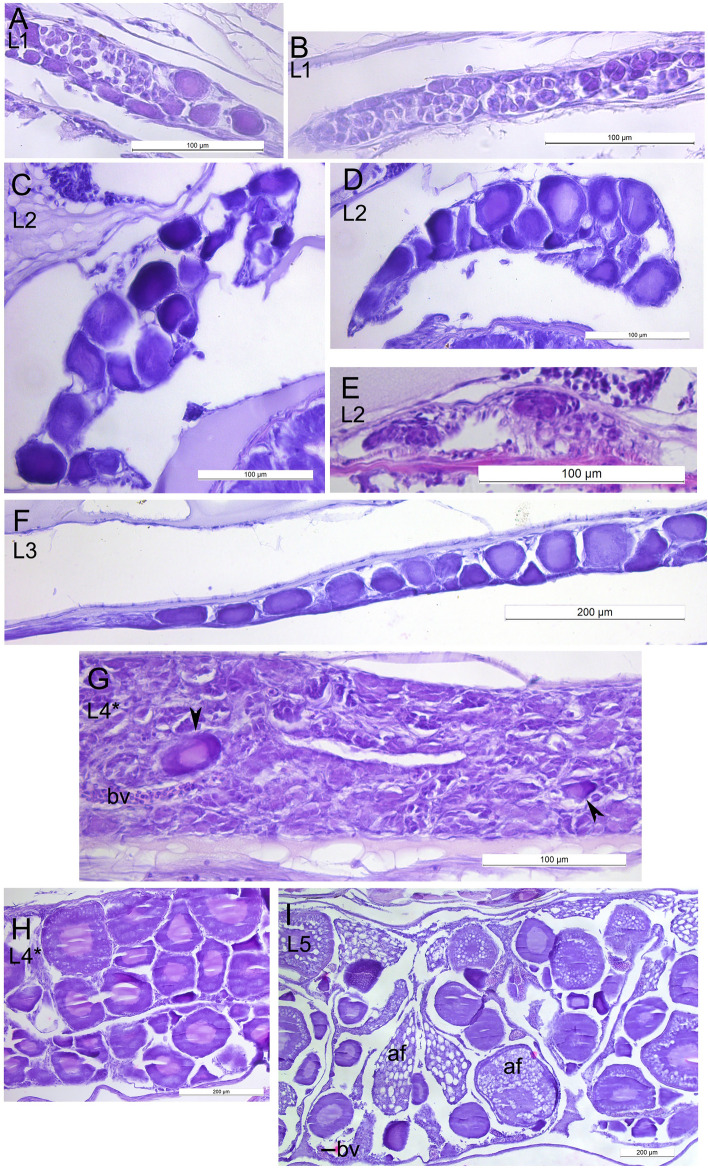
The state of gonads of *Leptoclinus maculatus* during postlarval development from the L2 to L5 stages. (**A**) Presumable ovary at the L1 stage. (**B**) Presumable testis at the L1 stage. (**C,D**) Ovary with previtellogenic oocytes. Transverse sections at the two levels along AP axis of the same specimen at the L2 stage. (**E**) Presumable developing testis. Transverse section. L2 stage. (**F**) The ovary with previtellogenic oocytes at the L3 stage. Sagittal section. (**G**) Presumable testis at the L4* stage. Arrowheads point to previtellogenic oocytes (anomaly). Sagittal section. (**H**) Ovary at the L4* stage. Particular oocytes contain cortical alveoli, and, probably, lipid inclusions. Sagittal section. (**I**) Ovary at the L5 stage. Cortical alveoli and lipid inclusions are concentrated at the periphery of oocytes. Atretic follicles are present. *af *atretic follicle, *bv *blood vessel.

The organizations of the developing gonads of *L. maculatus* are shown in Fig. 7. The ovary of postlarvae at the L1, L2 and L3 stages (Fig. 7A,C,D,F) contained previtellogenic oocytes. Presumable developing testis were found at the L1 and L2 stages (Fig. 7B,E). Interestingly, the gonad of the male at the L4* contained several previtellogenic oocytes, which is probably pathological (Fig. 7G). At the stage L4* cortical alveoli and, probably, lipids were accumulated in particular oocytes (Fig. 7H). In the female at the L5 the oocytes with cortical granules and lipids at the periphery were present (Fig. 7I). Notably, numerous atretic follicles at the different stages of degeneration were observed.

## Discussion

### Lipid sac

The lipid sac is a lipid storage organ of the *L. maculatus* postlarvae located in the body cavity from the pectoral fins to the anus and is not directly connected with the organs of the digestive tract. The structure of the lipid sac was first described in^4^ followed by a series of studies^2,3,5,18^. Herein the structural organization of the lipid sac at the L1 stage was first compared to that at later stages. Here we put forward that the multinucleate cells and syncytium within the connective tissue under the gut found at L1 stage are the initial stages of the lipid sac formation. Alternatively, these big cells with large nuclei could be the remnants of the yolk syncytial layer (YSL). The YSL is a multinucleate syncytium, which is formed during the blastula period. The YSL is included into the yolk complex that, in turn, becomes a part of the yolk sac (along with mesodermal and ectodermal derivatives). However, there are no morphological signs of the YSL programmed death^19^ at the L1 stage of the postlarvae. Additionally, the organization of the connective tissue characteristic of the lipid sac speaks against this proposition.

It was shown that the lipid sac is fully formed at the L2 stage, and its lipid compartments are gradually filled with lipids during the postlarval development. The lipids are transported from the blood to the lipid sac units. The wall of each unit consists of the connective tissue (up to several rows), the blood vessels, and the LSL. The blood vessels form the network of polygons. The multinucleate LSL interacts with the lipid vacuoles directly and plays a significant role in the lipid transport. The lipid sac is depleted (the volume of its units decreases, while the LSL thickness increases) at the L5 stage when the fish switches to benthivorous feeding upon entering the benthic habitat.

The size of the LSL nuclei increased significantly by the L4* stage of the postlarvae which most probably reflects the polyploidization linked to the onset of metabolic processes during switching to the benthic habitat. Notably, the polyploidization is not the only reason for the increase in the size of the nuclei^20,21^. The formation of temporary structures is more diverse and plastic than that of definitive organs; however, many of them share several common features. Numerous “extraembryonic” systems of both invertebrates and vertebrates are syncytial and/or have enlarged polyploid nuclei^19,22,23^. The nuclei in the LSL are generally regular-shaped, unlike the nuclei of some other transient structures^19,22^. The multinucleate LSL also shares some common features with the YSL, such as the complication of the shape and an increase in the linear dimensions of the nuclei during the development, the different content of the eu- and heterochromatin in the nuclei, and the intensification of the symplast functioning s during the transitional periods^2,3^. Both LSL and YSL perform trophic function; however, the YSL utilizes the nutrients of the maternal origin. On the contrary, the lipid sac accumulates the lipids obtained with active, exogenous feeding and utilizes them at the transitional stages. The YSL also performs morphogenetic and immune functions, but there is no data on these functions of LSL^22,24,25^. LSN are smaller and have less complex shapes than the yolk syncytial nuclei^26,27^.

Given the presence of the yolk in the oocytes of *L. maculatus*^18^ and the multiple vital functions of the YSL during the embryogenesis and postembryogenesis of fish, there is no doubt that embryos and larvae have the YSL. The yolk complex was not found in any specimen at the L1 postlarval stage. Thus, the interaction of these structures is improbable. The YSL is a specialized structure shown to finally undergo a programmed death, and most probably it does not contribute to the LSL formation^27^.

### Thyroid

The following thyroid patterns of Teleostei were defined^28^: a compact gland (Osteoglossomorphs, some Acantomorph families); a diffuse gland with the islets of follicles near the ventral aorta; a diffuse gland with the follicles located in the branchial region and the ectopic follicles in the cephalic kidneys, in the choroid region and along large blood vessels; the follicles are forming organized lobes; and a compact gland included in a blood sinus. The thyroid gland of *L. maculatus* is diffuse, and its follicles were found only in the branchial region near the ventral aorta at the studied L1–L3 stages. This is the second pattern, which is the most common in Teleostei^28^. The thyroid tissue of the fish species from the Barents Sea (cod *Gadus morhua morhua* (L.), haddock *Melanogrammus aeglefinus* (L.), capelin *Mallotus vilosus* L.) has in the form of follicle clusters around the ventral aorta and gill arteries^29^. The current study shows that the diameters (short and long) of thyroid follicles significantly increased from the L1 to the L2 of the daubed shanny. Most follicles are discrete; however, some of them are conjoined, doubled, or organized in chains. The similar chains of follicles were present in *Odontesthes bonariensis*^30^, *Rachycentron canadum*^31^. This organization most probably indicates the continued folliculogenesis^32^. The absence of reabsorption vesicles (“vacuoles”) at the periphery of the colloid may point to the low thyroid gland activity; however, the biochemical study is necessary to determine the hormone levels^33,34^. It is known that thyroid hormones play a vital role during the metamorphosis of fish and steroid hormones can modulate their effects^35,36^. The estradiol content in the muscles during the metamorphosis of the *L. maculatus* from the fjords of Spitsbergen was studied by Nemova et al.^37^. Studying the thyroid function in the fish of the Arctic seas can help reveal the mechanisms of stimulation and inhibition of certain vital processes, including the development, growth, and reproduction, which are orchestrated by thyroid hormones or their analogs^29^.

### Heart

The teleost heart shows a considerable species variability in the organization and functioning of its components, including the valves^38,39^. The heart of *L. maculatus* consisted of six compartments within the pericardium, from anterior to posterior: bulbus arteriosus (BA), conus arteriosus, ventricle, AV segment (between the atrium and the ventricle), atrium, sinus venosus. The shape, structure, and ultrastructure of BA vary widely among the teleost species^38–41^. The BA maintains a steady blood flow into the gill system through heart contractions. The BA wall of *L. maculatus* is not pigmented compared with those of *Gasterosteus aculeatus* and *Pungitius pungitius,* although their structure is similar^42^. The inner surface has relatively short and simple-shaped longitudinal ridges similar to those of zebrafish and different from those of *Thunnus albacares*^40,43^. The important difference between the BA of *L. maculatus* and zebrafish is the absence of blood vessels. When compared to the BA of the Antarctic teleosts, it appears more similar to the BA of the white-blooded *Chionodraco hamatus* than to that of the red-blooded *Trematomus bernacchii*. The daubed shanny has a conus arteriosus in the heart. The conus arteriosus has not been lost in the evolution of Teleostei and is a distinct segment interposed between the ventricle and the BA, being formed by the compact myocardium^44^. The conus arteriosus of the Bony fishes generally has one row of valves of two pocket-like leaflets and supporting sinus. In most teleosts, each conal valve leaflet consists of a stout proximal body and a flap-like distal region^38,39,44^. The daubed shanny postlarvae also have this organization of the conal valves.

The ventricular myocardium of *L. maculatus* was entirely trabeculated and lacks compacta. Thus, the heart of *L. maculatus* belongs to the type-I, which is the most common in Bony fishes. In *C. hamatus* the ventricle was entirely trabeculated^38,39,45,46^. However, the organization and ventricle type can change during the ontogeny. For example, compacta appears during the postembryonic development of *Arapaima gigas*^47^. The atrioventricular AV segment consists of a compact myocardial ring surrounded by a connective tissue ring that is species-specific. Additionally, it contains a complex network of nerves and neurons. The AV ring supports the AV valves, which contain an atrial fibrosa and variable amounts of cells, collagen, and elastin^48^. The atrium of the bony fishes consists of an external myocardial ring and a complex trabecular network. In *L. maculatus* the thin atrium is located dorsally to the ventricle. The sinus venosus collects all the venous blood via the ducts of Cuvier and the hepatic veins. The amount of myocardium was also variable. In *L. maculatus* the sinus venosus is thin-walled. The sinoatrial valve was not observed. Previous studies showed that it may be absent^39^. Thus, the heart organization of the daubed shanny is common for Teleostei, but the specific features were also found.

### Blood

An interesting finding of this study was the absence of distinguishable red blood cells at the L1, L2, and L3 stages of the postlarvae similar to that in the Antarctic fish. The fish from the Chaenichthyidae family inhabiting cold waters of Antarctic seas (temperature < 2 °C) also have no erythrocytes or hemoglobin in their blood^49^. It was shown that β-globin genes and the genes of embryonic/juvenile globins are lost in the fish from the Chaenichthyidae family, and α1-globin-related DNA sequences are inactive in this lineage. The ability to produce myoglobin was further lost several times^50–52^. Cold waters are more oxygenated which creates conditions for the simplified breathing and the loss of hemoglobin value^53^. The respiration of fish from the Chaenichthyidae family is known to occur by the diffusion of oxygen through the capillaries of the skin or gills, which have a larger surface area than in other fish^54^. The availability of oxygen is higher in the pelagic zone than in the bottom layers of water due to the active mixing of water masses. Supposedly, the way of respiration in the pelagic daubed shanny postlarvae is the same as in the larvae of the Antarctic fish by the capillaries of the skin and gills; however, this requires additional research.

Numerous erythrocytes were found only on the L4* stage. Oxygen requirements are known to increase during the metamorphosis, with active growth and the transition of postlarvae to the benthic lifestyle^55^. The acceleration of the fish growth rate of also correlates with the increased hemoglobin level in their body. In most vertebrates, the shifts in globin expression patterns occur during development. The changes in the expression of globins coincide with the shift of the anatomical site of hematopoiesis; however, as shown in *Oryzias latipes*, specific globins can be involved in both primitive and definitive hematopoiesis^56–59^. In the teleost *Paralichthys olivaceus* larval erythrocytes differ morphologically from the juvenile ones^60^. The number of erythrocytes in fish varies widely, primarily depending on the mobility of the fish. The pelagic larvae of the daubed shanny (L1–L3 stages) passively swim, also due to the lipid sac, which maintains their buoyancy, whereas during the life at the bottom, they need to move more actively looking for food. In the work^3^, an increase in the functional load on the muscles of the *L. maculatus* benthic early juveniles was established, which is associated with the search for food, especially during the polar night.

### Digestive system

The observed teething in the *L. maculatus* from the L1 stage probably indicates the process of the postlarval preparation for the transition to feeding on calanoid copepods at the L2 stage^6,13^. The taste buds in *L. maculatus* were already present on the L1 stage and their number increases at the L2 stage that may be related to selecting its main food item—lipid-rich zooplankton *Calanus* spp.^3,4,6,13^. The taste buds have been similar in structure to those of zebrafish and *Hucho taimen*^61,62^. *L. maculatus* had a stratified esophageal epithelium that is probably less common in the larvae of marine fishes than a columnar epithelium^63,64^. The stomach with prominent glandular and aglandular regions was formed by the L4* stage. The gastric glands appear by the L4* stage indicating the end of the larval mode of digestion. *L. maculatus* can be defined as altrical because its stomach develops at metamorphosis^65^. The adipose tissue in the pancreas also appears at this stage. We assume that it is defined by the near-bottom lifestyle at the L4* stage and switching to feeding on polychaetas, and small crustaceans as adults^6,13,14^.

### Liver

Small lipid inclusions found in the liver of postlarvae at the L1 stage indicate, among other characteristics, continuing exogenous feeding. They were more prominent at the L2 and the L3 stages and decreased by the L4* stage that may be related to the shift from the postlarval nutrition spectrum to that of adult demersal fish. The localization and appearance of the lipid droplets in the liver also changes depending on the season; for mature individuals, the histomorphological state of the liver is closely related to the reproductive period^5^.

### Gonads

The ovaries with previtellogenic oocytes and presumably male gonads were already observed at the L1 stage. This allows us to carefully propose that *L. maculatus* is a differentiated gonochoristic. The secondary growth phase, or previtellogenesis, is divided into two periods: the formation of cortical alveoli and the formation of lipid globules^5,66^. The cortical alveoli and, probably, the lipid inclusions in the oocytes were first observed at the L4 stage. An increase in the size and number of the cortical alveoli containing hydrolytic enzymes in the oocyte is necessary for the optimal functioning of enzymes. These structures forming in the early stages of development can later play an important role in fertilization^5^. Therefore, the oocytes in the ovaries of the postlarvae are in the secondary growth phase, previtellogenesis, for a long time (for 4–5 years) while the formation of the cortical alveoli begins only during the transitional stage at the L4, and the L4* stages with the detection of small lipid droplets. The first atretic follicles appear at the L5 stage, which may indicate the beginning of the maturation period of the gonads. The seasonal characteristics of lipid accumulation, as well as the changes in the gonads during the oogenesis of an adult *L. maculatus* have been histologically described in detail in our earlier studies^5,18^.

## Conclusion

*Leptoclinus maculatus* is characterized by a complex life cycle, with extended pelagic postlarval development accompanied by multiple biochemical and morphological ontogenetic changes^1,3,4^. In this study we provided histological descriptions of the organs and tissues of the fish postlarvae.

The follicles in the thyroid gland of *L. maculatus* are located diffusely in the branchial region near the ventral aorta at all the postlarval stages. We suppose that the thyroid activity in the postlarvae in the pelagic period is low due to the absence of the reabsorption vesicles (“vacuoles”) at the periphery of the colloid. The heart of the daubed shanny has the structure typical of most teleost fishes, but also exhibits a number of species-specific features (notably the bulbus arteriosus is not pigmented).

The lipid sac is a unique temporary feature of *L. maculatus* postlarvae in the Arctic serving as an adaptation to seasonal changes in food availability and low temperature^1,3,6^. The current work revealed the structure of the LS on the L1 stage (the youngest). Our first data allows for the careful proposition, that the LSL of the lipid sac is formed from the multinucleate cells located within the connective tissue under the gut. Our results show that the transition of the postlarvae from the pelagic lifestyle to the benthic one at L4* stage is accompanied by the decrease in the volume of units, but the increase in the LSL thickness.

Notably, the transition of the daubed shanny postlarvae to the benthic lifestyle is connected not only with the depletion of the lipid sac as a distinctive ontogenetic feature, but also with other adaptive changes in its body established in this study:

1. The absence of distinguishable red blood cells in the pelagic postlarvae of the daubed shanny reveals similarities with the Antarctic fish (Chaenichthyidae family). Numerous erythrocytes appear in the blood at the L4* postlarval stage possibly due to the increased physical activity more oxygen during its benthic period of life.
2. The decreasing size of the lipid droplets in the hepatocytes at the L4* stage may be related to the transition to the benthic lifestyle and therefore to the nutrition spectrum typical of adults.
3. The stomach of the daubed shanny postlarvae is formed by the L4* stage, and it has prominent glandular and aglandular regions.
4. The formation of the cortical alveoli begins only at the L4* transitional stages when small lipid droplets in the oocytes can be detected in the ovaries. At the early juvenile L5 stage, the first atretic follicles appear indicating the beginning of the gonad maturation.

## Supplementary Information

Supplementary Information.

## Data Availability

All data generated or analyzed during this study are included in this published article (and its Supplementary Materials).
