# Supplementary material for: Ontogenetic changes in the body structure of the Arctic fish Leptoclinus maculatus
Source: Sci Rep. 2023 Mar 6;13:3688. doi: 10.1038/s41598-023-30251-5 (PMC9988964; doi:10.1038/s41598-023-30251-5)
Supplement: Supplementary file 1 — Supplementary Information. [file 41598_2023_30251_MOESM1_ESM.pdf]

Supplementary materials

I. Figures

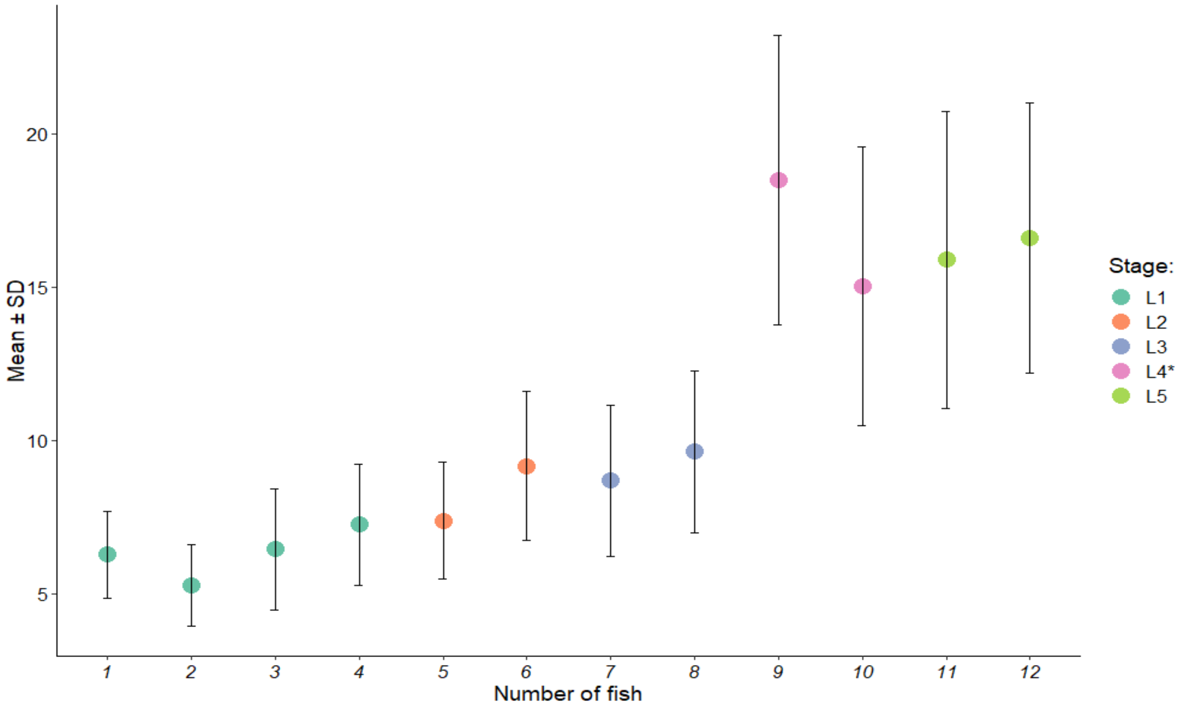

Supplementary Figure S1. Length of nuclei of the lipid sac. Ranges of the standard deviation (sd) of the variance for each fish

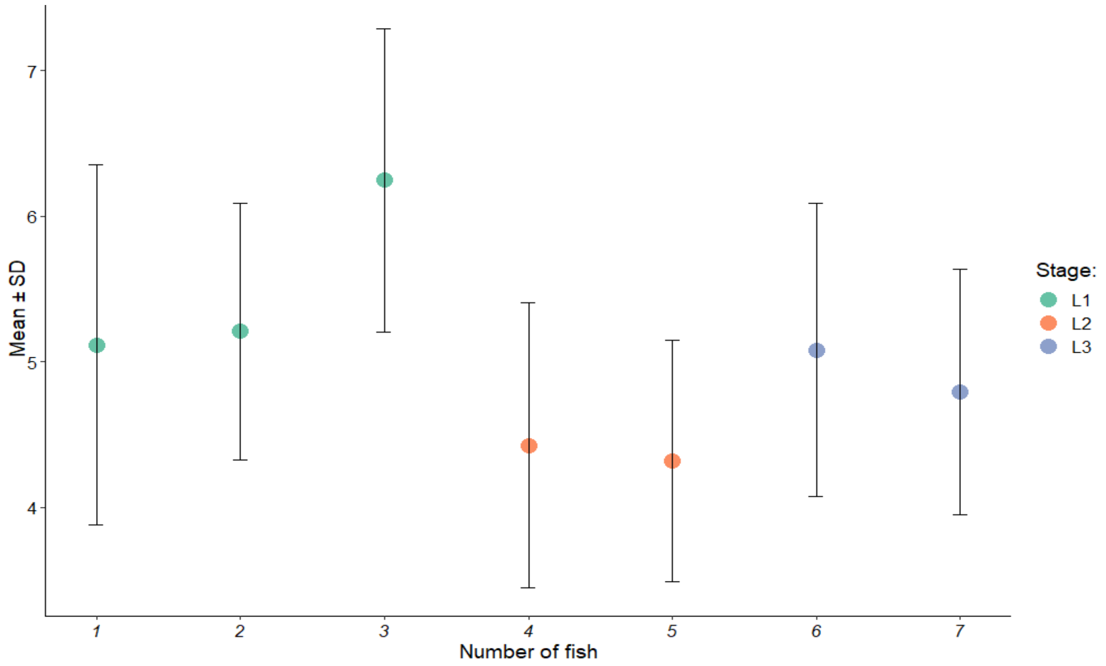

Supplementary Figure S2. Height of thyrocytes in thyroid gland. Ranges of the standard deviation (sd) of the variance for each fish

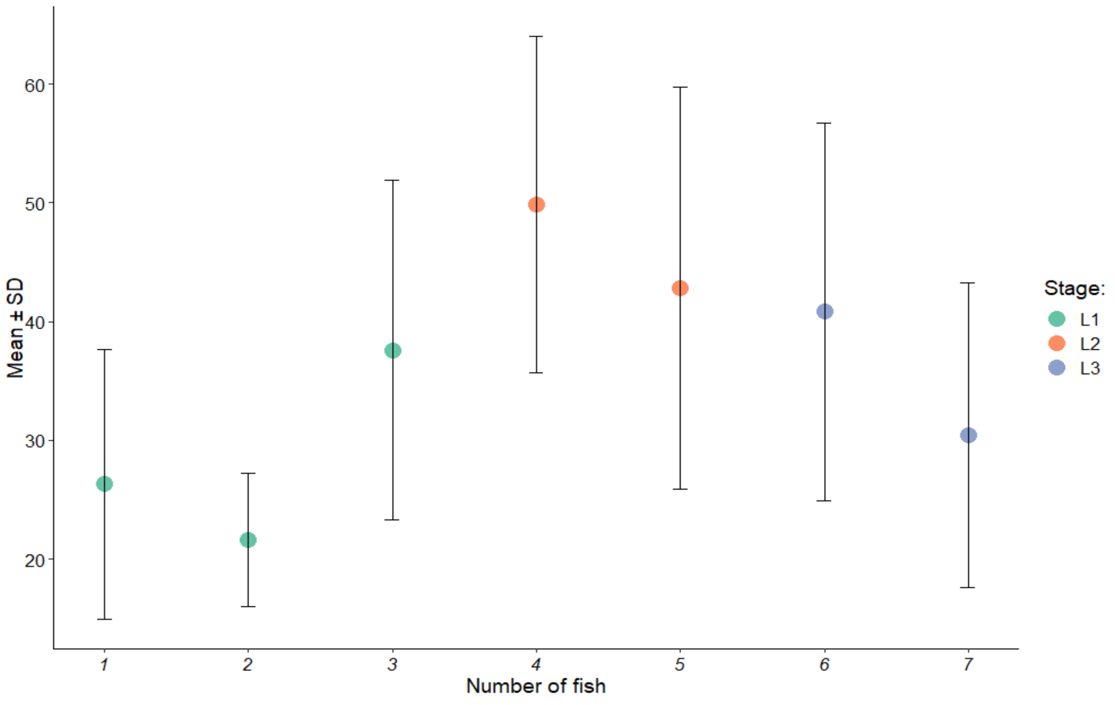

Supplementary Figure S3. Short diameter of follicles in thyroid gland. Ranges of the standard deviation (sd) of the variance for each fish

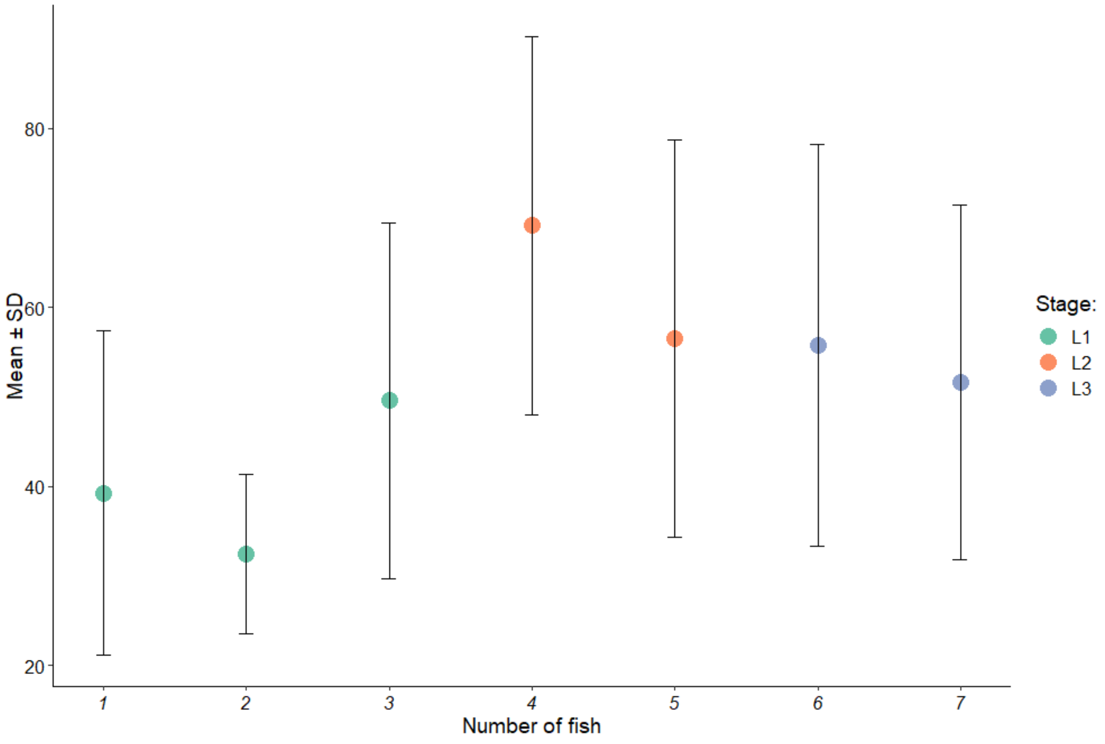

Supplementary Figure S4. Long diameter of follicles in thyroid gland. Ranges of the standard deviation (sd) of the variance for each fish

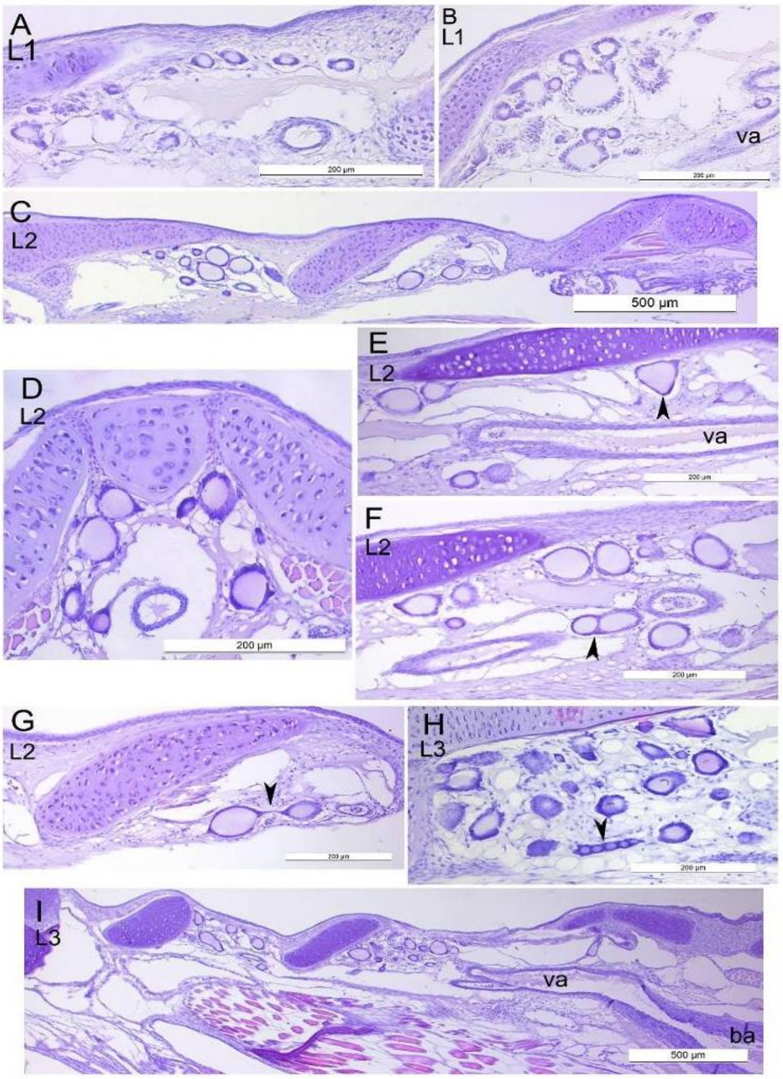

Supplementary Figure S5. The thyroid gland of *Leptoclinus maculatus* postlarvae at the L2 and L3 stages of development. (A-C, E-I) sagittal sections. (A) Small scattered follicles in the branchial region at the L1 stage. (B) The larger follicles. Note the follicle connected with the four other ones. (C) General view of the branchial region with scattered thyroidal follicles. The L2 stage. (D) Transverse section of the branchial region with thyroidal follicles at the L2 stage. (E) The arrowhead points to the triangular follicle at the L2 stage. (F) The arrowhead shows the doubled follicle at the L2 stage. (G) An arrowhead indicates the two interconnected follicles at the L2 stage. (H) The arrowhead shows the chain of five small follicles in the individual at the L3 stage. (I) The general view of branchial region, heart outflow tract and numerous scattered thyroidal follicles in the specimen at the L3 stage. Notes: ba - bulbus arteriosus; va - ventral aorta

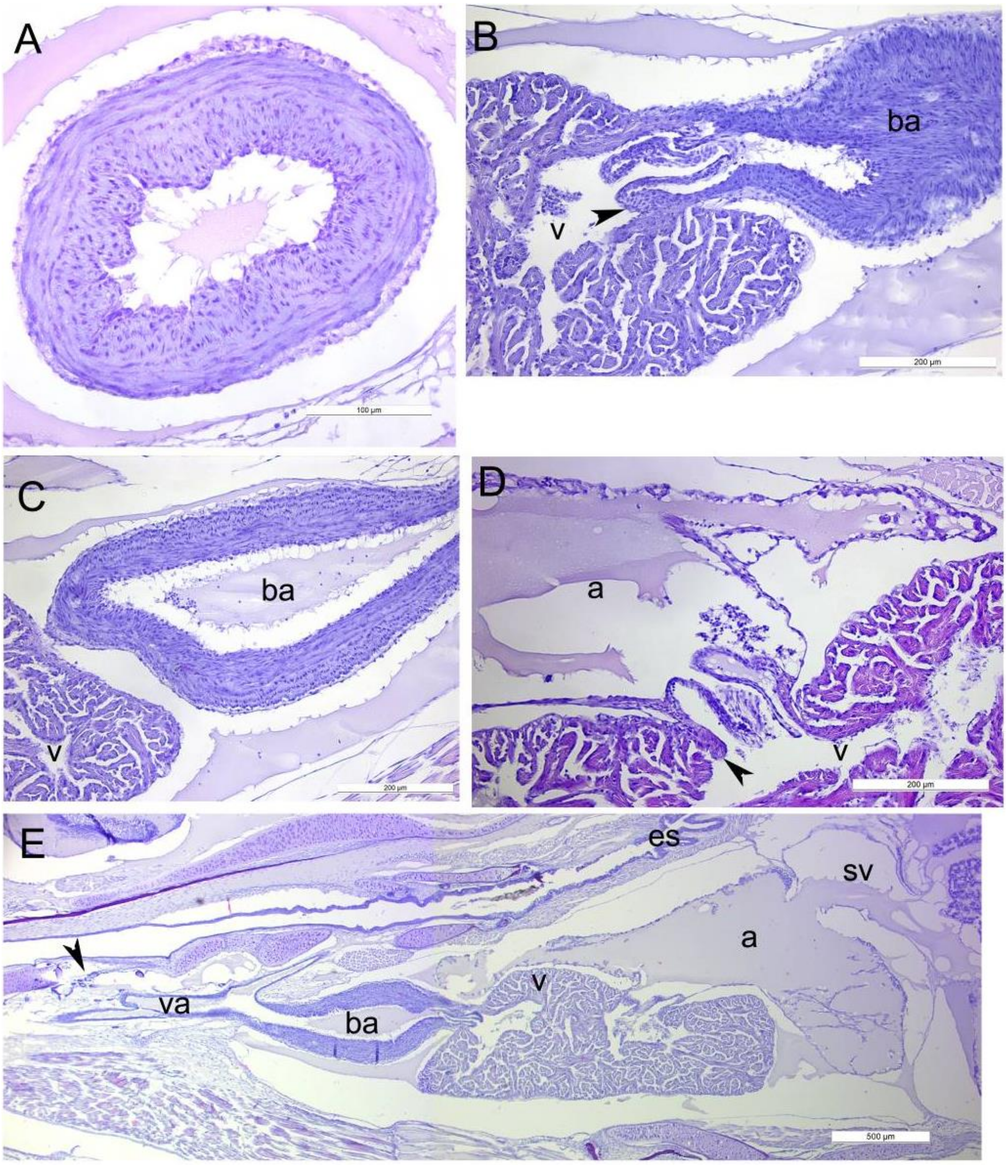

Supplementary Figure S6. The heart organization of *Leptoclinus maculatus* postlarvae at the L2 and L3 stages. (A) Transverse section of the bulbus arteriosus at the L2 stage. (B) Sagittal section of the conal valve. The arrowhead points to the leaflet with stout proximal body at the L2 stage. (C) Sagittal section of the bulbus arteriosus at the L2 stage. (D) Sagittal section of the AV valve. Arrowhead points to the myocardial ring. (E) The general view of the heart of postlarvae at the L3 stage. Sagittal section. Arrowhead indicates the thyroidal follicles. Notes: v – ventricle; ba - bulbus arteriosus; a - atrium; es - esophagus; va - ventral aorta; sv - sinus venosus.

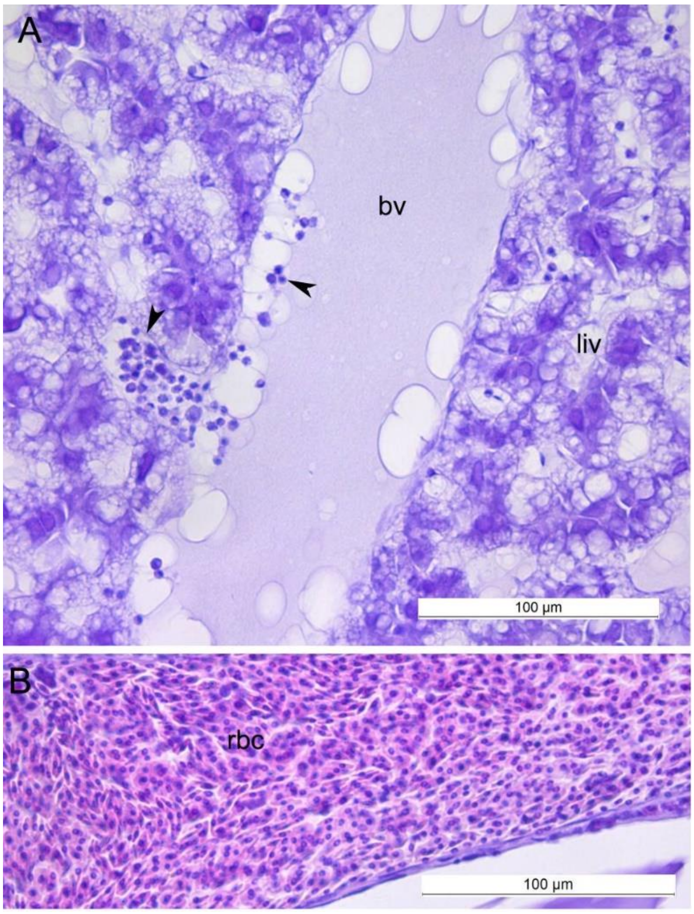

Supplementary Figure S7. Comparison of blood vessels of *Leptoclinus maculatus* postlarvae at the L2 and L5 stages. (A) The blood vessel in the liver. No cells with morphology of erythrocytes are found (arrowheads). (B) The vessel in the ventral region. Numerous red blood cells. Notes: bv - blood vessel; liv - liver; rbc - red blood cells.

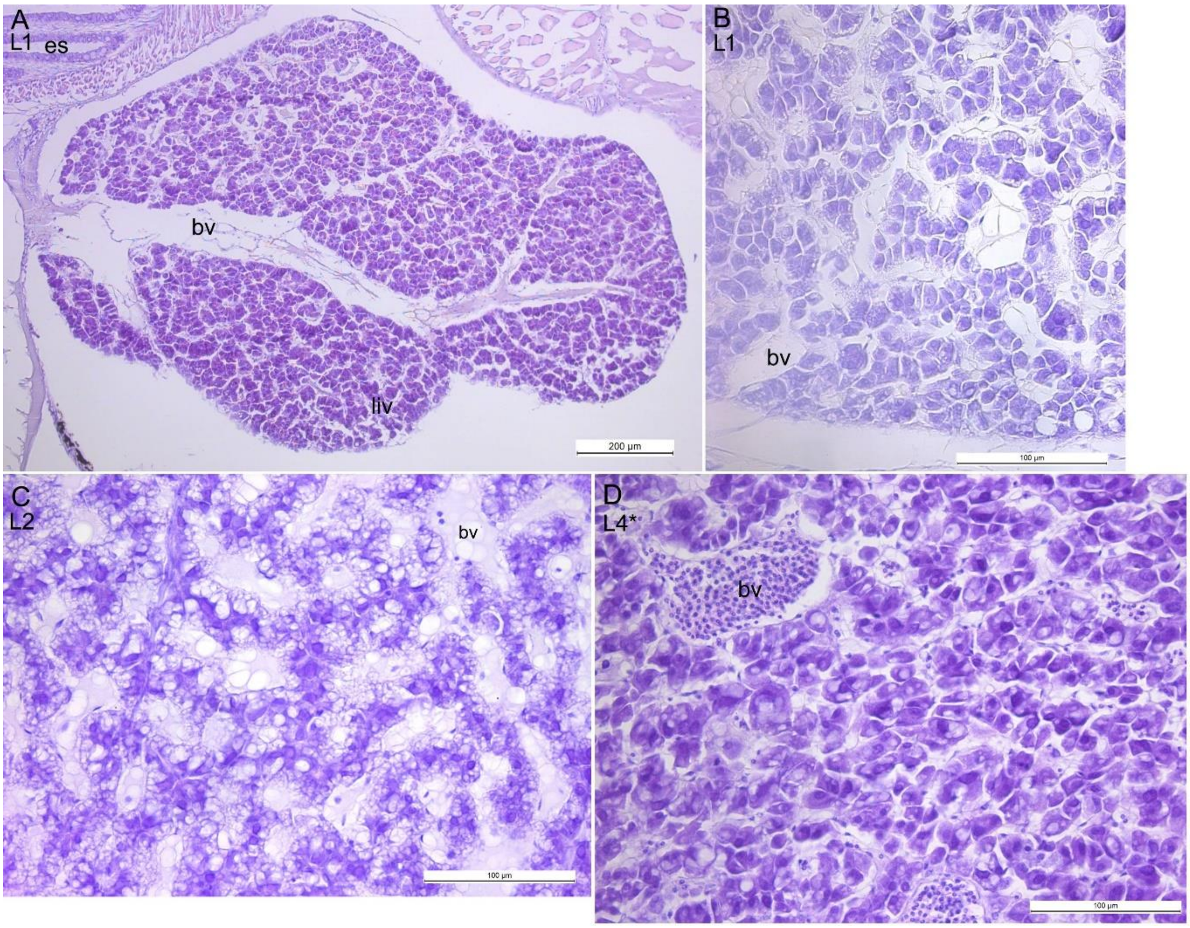

Supplementary Figure S8. The liver organization of *Leptoclinus maculatus* during postlarval development (from L1 to L4* stage). (A) General view of the liver with liver sinusoids at the L1 stage. (B) Liver at the L1 stage. (C) Liver at the L2 stage (D) Liver at the L4* stage. Notes: bv - blood vessels; es - esophagus, liv - liver.

II. Results

Statistical analysis report

1. Lipid sac

LRT for random effects (Fish)

Data: Lipid sac_the length of nuclei

Models:

mod2: Length ~ Stage + (1 | Fish)

mod1: Length ~ Stage + (1 + Stage | Fish)

	npar	AIC	BIC	logLik	deviance	Chisq	Df	Pr(>Chisq)
mod2	7	4906.0	4939.9	-2446.0	4892.0			
mod1	21	4930.3	5031.9	-2444.2	4888.3	3.6727	14	**0.9971**

Conclusion: The length of the nuclei in the lipid sac in different individuals does not depend on the stage of development.

LRT for fixed effects (Stage)

Data: Lipid sac_the length of nuclei

Models:

mod4: Length ~ 1 + (1 + Stage | Fish)

mod3: Length ~ Stage + (1 + Stage | Fish)

	npar	AIC	BIC	logLik	deviance	Chisq	Df	Pr(>Chisq)
mod4	17	4956.0	5038.3	-2461	4922.0			
mod3	21	4932.1	5033.7	-2445	4890.1	31.956	4	**1.954e-06 *****

Signif. codes: 0 ‘***’ 0.001 ‘**’ 0.01 ‘*’ 0.05 ‘.’ 0.1 ‘ ’ 1

Conclusion: The length of the nuclei in the lipid sac depends on the stage of development.

2. Thyrocytes

LRT for random effects (Fish)

Data: Thyrocytes height

Models:

mod2: Height ~ Stage + (1 | Fish)

mod1: Height ~ Stage + (1 + Stage | Fish)

	npar	AIC	BIC	logLik	deviance	Chisq	Df	Pr(>Chisq)
mod2	5	544.51	560.51	-267.26	534.51			
mod1	10	551.86	583.85	-265.93	531.86	2.6527	5	**0.7533**

Conclusion: The length of the thyrocytes in different individuals does not depend on the stage of development.

LRT for fixed effects (Stage)

Data: Thyrocytes height

Models:

mod4: Height ~ 1 + (1 + Stage | Fish)

mod3: Height ~ Stage + (1 + Stage | Fish)

	npar	AIC	BIC	logLik	deviance	Chisq	Df	Pr(>Chisq)
mod4	8	551.87	577.46	-267.93	535.87			
mod3	10	548.32	580.31	-264.16	528.32	7.5432	2	**0.02302 ***

Signif. codes: 0 ‘***’ 0.001 ‘**’ 0.01 ‘*’ 0.05 ‘.’ 0.1 ‘ ’ 1

Conclusion: The length of the thyrocytes depends on the stage of development.

3. **Follicles**

a) Short Diameter

LRT for random effects (Fish)

Data: follicles diameters

Models:

mod2: ShortD ~ Stage + (1 | Fish)

mod1: ShortD ~ Stage + (1 + Stage | Fish)

	npar	AIC	BIC	logLik	deviance	Chisq	Df	Pr(>Chisq)
mod2	5	2464.5	2483.2	-1227.3	2454.5			
mod1	10	2474.2	2511.5	-1227.1	2454.2	0.3132	5	**0.9974**

Conclusion: The short diameter of follicles in different individuals does not depend on the stage of development.

LRT for fixed effects (Stage)

Data: follicles diameters

Models:

mod4: ShortD ~ 1 + (1 + Stage | Fish)

mod3: ShortD ~ Stage + (1 + Stage | Fish)

	npar	AIC	BIC	logLik	deviance	Chisq	Df	Pr(>Chisq)
mod4	8	2490.5	2520.4	-1237.3	2474.5			
mod3	10	2487.7	2525.0	-1233.8	2467.7	6.8525	2	**0.03251 ***

Signif. codes: 0 ‘***’ 0.001 ‘**’ 0.01 ‘*’ 0.05 ‘.’ 0.1 ‘ ’ 1

Conclusion: The short diameter of the follicles depends on the stage of development.

b) Long Diameter

LRT for random effects (Fish)

Data: follicles diameters

Models:

mod2: LongD ~ Stage + (1 | Fish)

mod1: LongD ~ Stage + (1 + Stage | Fish)

	npar	AIC	BIC	logLik	deviance	Chisq	Df	Pr(>Chisq)
mod2	5	2689.3	2708.0	-1339.7	2679.3			
mod1	10	2698.1	2735.4	-1339.1	2678.1	1.1884	5	**0.946**

Conclusion: The long diameter of the follicles in different individuals does not depend on the stage of development.

LRT for fixed effects (Stage)

Data: follicles diameters

Models:

mod4: LongD ~ 1 + (1 + Stage | Fish)

mod3: LongD ~ Stage + (1 + Stage | Fish)

	npar	AIC	BIC	logLik	deviance	Chisq	Df	Pr(>Chisq)
mod4	8	2714.5	2744.3	-1349.2	2698.5			
mod3	10	2711.7	2749.0	-1345.8	2691.7	6.805	2	**0.03329 ***

Signif. codes: 0 ‘***’ 0.001 ‘**’ 0.01 ‘*’ 0.05 ‘.’ 0.1 ‘ ’ 1

Conclusion: The long diameter of the follicles depends on the stage of development.
